# Rationale, objectives and design of PURE, a prospective registry of patients with moderate to severe chronic plaque psoriasis in Canada and Latin America

**DOI:** 10.1186/s12895-019-0087-3

**Published:** 2019-06-21

**Authors:** Kim A. Papp, Melinda Gooderham, Jennifer Beecker, Charles W. Lynde, Isabelle Delorme, Ignacio Dei-Cas, Lorne Albrecht, Emmanouil Rampakakis, John S. Sampalis, Antonio Vieira, Shamiza Hussein, Olivier Chambenoit, Lenka Rihakova

**Affiliations:** 1 Papp Clinical Research and Probity Medical Research, Waterloo, ON Canada; 2 SKiN Center for Dermatology, Queen’s University and Probity Medical Research, Peterborough, ON Canada; 3 Division of Dermatology, University of Ottawa, Ottawa, ON Canada; 4 Lynde Institute for Dermatology, University of Toronto and Probity Medical Research, Markham, ON Canada; 5 Dr. Isabelle Delorm Inc, Drummondville, QC Canada; 6 Facultad de Medicina de la Universidad de Buenos Aires, Buenos Aires, Argentina; 7 Enverus Medical Research, University of British Columbia and Probity Medical Research, Surrey, BC Canada; 8 JSS Medical Research, Montreal, Quebec Canada; 9 Novartis Pharmaceuticals Canada Inc., Dorval, QC Canada; 10 Novartis Pharmaceuticals Corporation, East Hanover, NJ USA

**Keywords:** Real-world, Observational, Non-interventional, Registry, Safety, Effectiveness, Psoriasis, Secukinumab, Biologic, Systemic therapy

## Abstract

**Background:**

Treatment options for the management of moderate to severe plaque psoriasis include phototherapy, oral systemic agents, and biologic therapy. Secukinumab, a fully human monoclonal antibody that selectively targets IL-17A, is the first IL-17 antagonist approved for this patient population. Long-term observational data are required for establishing the true population-based benefit-risk ratio of approved treatments. PURE is a multinational registry that will assess the real-world safety and effectiveness of secukinumab and other approved therapies in the management of patients with moderate to severe psoriasis.

**Methods:**

This is a multinational (Canadian and Latin American), prospective, observational study of adult patients with moderate to severe psoriasis that initiate treatment with secukinumab or other approved therapies as per local standard of care. A total of 2500 patients (1250 per cohort) will be recruited in the practices of hospital and community dermatologists. Decision regarding treatment must have been reached prior to and independent of patient enrollment in the study. The study includes a 5-year follow-up with recommended assessments at Baseline, 3 and 6 months post-Baseline, and every 6 months thereafter. The primary objective of the study is safety. Secondary outcome measures relate to effectiveness (Investigator’s Global Assessment -IGA mod 2011-, Psoriasis Areas and Severity Index, Body Surface Area), patient reported outcomes (Dermatology Life Quality Index, Work Productivity and Activity Impairment Questionnaire, Hospital Anxiety and Depression Scale, Psoriasis Epidemiology Screening Tool, Psoriasis Symptom Diary, and Treatment Satisfaction Questionnaire), and healthcare resource utilization.

**Discussion:**

This is the first observational study in Canada and Latin America assessing the real-world safety and effectiveness of secukinumab in the management of moderate to severe psoriasis. The extensive clinical, patient-reported and health economic outcomes collected will allow the comprehensive evaluation of this new treatment in comparison to other approved therapies.

**Trial registration:**

ClinicalTrials.gov Identifier: NCT02786186; date of registration: May 30, 2016.

## Background

Psoriasis is a chronic, immunologically-mediated dermatosis that is estimated to affect 2–3% of the Canadian population [1]. In Latin America, prevalence rates have been estimated to range between 1 and 3% though the actual prevalence is unknown. Chronic plaque psoriasis is the most frequent clinical presentation, and accounts for 90% of all cases. Along with physical discomfort, psoriasis is associated with depression, sexual impairment, social stigmatization and reduced work productivity [1, 2]. Up to 15.5% impairment in work productivity and 23.7% impairment in total activity have been reported to be associated with psoriasis [2]. Consequently, although generally non-fatal, this disease incurs a significant impact on quality of life both physically and psychosocially [1, 3], with reports of reduction in physical and mental functioning comparable to that seen in other chronic diseases [4].

In recent years, the attention paid to psoriasis-related comorbidities has amplified, and a distinct pattern of chronic disorders was found to be significantly associated with psoriasis, including psoriatic arthritis, diabetes mellitus type II, arterial hypertension, hyperlipidemia, and coronary heart disease [5–7]. The combined presence of these conditions, together with obesity, known as the metabolic syndrome, is more prevalent in psoriasis patients than the general population [6, 8]. As awareness of the association of chronic inflammatory diseases and metabolic and vascular diseases increases, it has been suggested that the anti-inflammatory properties of psoriasis treatment modalities may provide therapeutic or preventive benefit related to reduction of cardiovascular- and metabolic-related risk in psoriasis patients.

According to the most recent Canadian Guidelines for the management of plaque psoriasis, moderate to severe psoriasis is distinguished from milder disease in that it is, or would be expected to be, refractory to topical monotherapy [9, 10]. Consequently, the dermatologist’s toolkit for the treatment of moderate to severe plaque psoriasis is broad, and the current Canadian psoriasis guidelines recommend the following therapeutic options: topical calcipotriol–betamethasone dipropionate combination ointment, phototherapy (UVA with psoralen and UVB), oral systemic agents (cyclosporine, methotrexate, and acitretin) and biologic therapy. Similar treatment guidelines have been published for Latin American countries [11–13]. Until 2015, the only biologic therapies approved for the treatment of moderate to severe plaque psoriasis were anti-TNF and IL-12/23 targeting agents. With the introduction of secukinumab, the first IL-17 antagonist approved for moderate to severe plaque psoriasis, the treatment armamentarium was further expanded.

Even though secukinumab was shown to be safe and effective in controlled clinical trials, its long-term safety and effectiveness in the real-world management of moderate to severe plaque psoriasis is yet to be shown. Furthermore, in light of the constantly evolving therapeutic environment, it is important to evaluate how the introduction of secukinumab affects medical decision making in terms of patient selection for treatment with each therapy. The purpose of this observational registry will be to describe the safety, long term effectiveness, and impact on quality of life of secukinumab and other indicated therapies administered to patients with moderate to severe chronic plaque psoriasis in a real-world setting.

## Methods / design

### Objectives

The overall aim of PURE is to provide real-world evidence regarding the safety and effectiveness of secukinumab or other indicated therapies, including locally approved systemic, phototherapy, or biologic therapy alone or in combination.

### Primary objective

To describe the long-term safety profile of secukinumab and other indicated therapies in patients with moderate to severe chronic plaque psoriasis.

### Secondary objectives

1. To describe the long-term effectiveness, including clinical and quality of life, of secukinumab and other indicated therapies in patients with moderate to severe chronic plaque psoriasis.
2. To describe the long-term impact on productivity of treatment with secukinumab and other indicated therapies in patients with moderate to severe chronic plaque psoriasis.
3. To describe the patient profile, with respect to demographics and clinical features, in patients with moderate to severe chronic plaque psoriasis treated with secukinumab and other indicated therapies.
4. To describe the rate of treatment discontinuation and durability of treatment in patients treated with secukinumab and other indicated therapies for moderate to severe chronic plaque psoriasis.
5. To describe the physical and mental health status of patients treated with secukinumab and other indicated therapies for moderate to severe chronic plaque psoriasis.
6. To describe satisfaction with treatment with secukinumab and other indicated therapies in patients with moderate to severe chronic plaque psoriasis.
7. To describe the socioeconomic impact of disease for patients treated with secukinumab and other indicated therapies in patients with moderate to severe chronic plaque psoriasis.

### Study design

PURE is a multinational, prospective, observational cohort study conducted in the practices of hospital and community dermatologists in Canada and Latin America (Argentina, Brazil, Costa Rica, Guatemala, Mexico, Panama) that treat patients with moderate to severe chronic plaque psoriasis. [14] Patients with moderate to severe chronic plaque psoriasis whom, prior to and independent of study enrollment, the treating physician has decided to treat with an indicated approved regimen as per standard of care in each participating country.

Patients are followed for up to 5 years from the time of enrollment. In accordance with the observational nature of the study, no assessment visits are imposed for the exclusive purpose of the study, however follow-up visits are recommended at 3 and 6 months post-baseline and every 6 months thereafter. Furthermore, any changes in the management of the patient including, but not limited to, changes in medication dose or frequency, termination of a medication or initiation of a new treatment is at the sole discretion of the treating physician and is to be recorded in the study data collection forms. At any time, patients may be withdrawn from the study by the treating physician or on the patient’s expressed choice.

There are two distinct patient cohorts in the study, those that initiate treatment with secukinumab and those that initiate treatment with other indicated and country-approved therapies as per local standard of care. Given the longitudinal nature of the study, it is anticipated that treatment cohort cross-over can occur. All patients will be followed in the study regardless of and after treatment cross-over, as long as they continue to fulfill the study inclusion and exclusion criteria. All analyses will be based on the intent-to-treat (ITT) principle even though treatment cohort may be considered as a time-dependent variable in some analyses.

### Patient population

In order to be included in the study, potentially eligible patients have to fulfill all of the following inclusion and none of the following exclusion criteria.

#### Inclusion criteria

1. Able to give written informed consent.
2. At least 18 years of age at time of informed consent signature.
3. Confirmed diagnosis of moderate to severe chronic plaque-type psoriasis diagnosed by a specialist and presence of moderate to severe psoriasis symptoms according to the physician’s clinical judgment at the time of recruitment.
4. Patient is initiating a treatment for psoriasis as per regional policy. This may include secukinumab, other biologics, systemic treatments, and phototherapy. Decision to treat with any of the above-mentioned treatments must have been reached prior to and independently of recruitment in the study.
5. Treatments are prescribed in accordance to the product monograph and regional regulatory and reimbursement policies.
6. Patient is able to understand and communicate with the investigator and comply with the requirements of the study.

### Exclusion criteria

1. Unwillingness or inability to comply with the study requirements
2. Participation in an interventional or non-interventional clinical trial, concurrently or within the last 30 days.

### Study variables

#### Baseline assessments

During the baseline visit, data on patient demographics (age, gender and race), employment, household income, health insurance, medical history and comorbidity are recorded. In addition, a brief physical examination with measurements of height, weight, blood pressure and pulse rate is conducted. The dates of onset of psoriasis symptoms and of psoriasis diagnosis, prior and current psoriasis therapies are also ascertained.

#### Outcome measures

The following outcome measures are to be assessed at each study visit:

- The incidence of adverse events (AEs).
- Investigator’s Global Assessment for General Psoriasis (IGA Mod 2011).
- Psoriasis Area and Severity Index (PASI).
- Body Surface Area (BSA).
- Dermatology Life Quality Index (DLQI).
- Work Productivity and Activity Impairment (WPAI) Questionnaire.
- Hospital Anxiety and Depression Scale (HADS).
- Psoriasis Epidemiology Screening Tool (PEST).
- Health Resources Utilization (HRU) Questionnaire.
- Psoriasis Symptom Diary (PSD).
- Treatment Satisfaction Questionnaire.

#### Outcome variables

The primary outcome variable will be the incidence of total AEs, total serious AEs (SAEs) and infections, as measured by the proportion of patients with at least one event, the mean number of events per patient, and the mean number of events per person-year of follow up. All adverse events will be mapped, based on the following hierarchy of exposures (in descending order of priority): [1] secukinumab, [2] other, [3] and non-biologic agents.

The following secondary outcome variables will be used:

1. Physician-Reported Outcomes:
  - Change in IGA from baseline to each visit.
  - Rate of change in IGA for the 5-year follow up duration of the study.
  - Proportion of patients achieving IGA ≤ 1 at any point in time.
  - Time to achieving IGA ≤ 1 during the study follow up period.
  - Durability of effect (IGA ≤ 1).
  - Proportion of patients achieving IGA = 0 at any point in time.
  - Time to achieving IGA = 0 during the study follow up period.
  - Durability of effect (IGA = 0).
  - Change in PASI from baseline to each visit.
  - Rate of change in PASI for the 5-year follow up duration of the study.
  - Proportion of patients achieving PASI 100, 90, 75 and 50 at any point in time.
  - Time to achieving PASI 100, 90, 75 and 50 during the study follow up period.
  - Durability of effect (PASI 100, 90, 75 and 50).
  - Change in BSA from baseline to each visit.
  - Rate of change in BSA for the 5-year follow up duration of the study.
2. Patient-Reported Outcomes:
  - Change in DLQI from baseline to each visit.
  - Rate of change in DLQI for the 5-year follow up duration of the study.
  - Proportion of patients achieving DLQI ≤1 at any point in time.
  - Time to achieving DLQI ≤1 during the study follow up period.
  - Durability of effect (DLQI ≤1).
  - Change in WPAI from baseline to each visit.
  - Rate of change in WPAI for the 5-year follow up duration of the study.
  - Change in the HADS score from baseline to each visit.
  - Rate of change in the HADS score for the 5-year follow up duration of the study.
  - Change in the PEST score from baseline to each visit.
  - Rate of change in the PEST score for the 5-year follow up duration of the study.
  - Distribution of responses to the HRU questionnaire at each visit.
  - Change in HRU from baseline to each visit.
  - Change in the PSD from baseline to each visit.
  - Rate of change in the PSD score for the 5-year follow up duration of the study.
  - Distribution of responses to the patient satisfaction question at each visit.
  - Change in patient satisfaction from baseline to each visit.
  - Proportion of patients adherent to treatment.
3. Other Outcomes:
  - Treatment discontinuation and durability.
    i. Proportion of patients discontinuing treatment.
    ii. Reasons for treatment discontinuation
    iii. Time to treatment discontinuation.
    iv. Prevalence and incidence of select comorbid conditions, with emphasis on comorbidities associated with psoriasis (e.g. psoriatic arthritis, diabetes, metabolic syndrome, cardiovascular disease)

### Statistical methods

#### Sample size considerations

This is an observational study aimed at describing the safety, effectiveness and patient reported outcomes in patients treated with secukinumab and other indicated approved therapies for moderate to severe chronic plaque psoriasis. Given the observational nature of the study, sample size considerations are based on the precision of measuring the primary outcome measure, namely the incidence of total adverse events (AEs), total serious AEs (SAEs) and infections, as measured with the width of the 95% confidence interval (CI).

With 1250 patients in the secukinumab cohort, and assuming an incidence rate of 60% for total AEs, the 95% CI will be 60% ± 3%, or 5% of the point estimate; for SAEs, assuming an incidence of 5%, a sample size of 1250 patients will yield a 95% CI of ±1.2% or 24% of the point estimate; for infections, assuming an incidence of 30%, a sample size of 1250 patients will produce a 95% CI of ±3%, or 10% of the point estimate. All within acceptable level of precision.

Although the primary aim of the study will be to describe the incidence of AEs in the two study cohorts, between cohort comparisons may be conducted for descriptive/exploratory purposes only without any a-priori hypothesis testing. With 1250 patients per cohort, the study will be able to detect with 90% power and 5% significance, an odds ratio of 1.30 for any adverse event, an odds ratio of 1.75 for SAEs, and an odds ratio of 1.35 for infections.

Approximately 80% are planned to be recruited from Canada and 20% from the participating Latin American countries.

### Statistical analyses

AEs and SAEs will be summarized for the two treatment cohorts using the total number of events, the total number of patients and percentage of patients who experienced at least one event within each body system and within each preferred term of the Medical Dictionary for Regulatory Activities (MedDRA); in these descriptive analyses, crossed-over patients (to either direction) will be described as a separate treatment cohort. Within each treatment cohort, patients experiencing the same event multiple times will be only counted once for the corresponding preferred term and body system. Relative risk/odds and incidence density rate ratio will be estimated to assess the difference between cohorts.

The levels of all physician-reported and patient-reported outcomes will be described for all visits by baseline treatment cohort; between-cohort differences will be assessed using the independent-samples t-test (or non-parametric alternative, as needed) for continuous variables and the chi-square test for categorical variables. In addition, the absolute changes in IGA, PASI, and BSA, will be described and the paired t-test will be conducted to assess the within-group change from baseline at each visit. Multivariate mixed models with repeated measures that may adjust for potential confounders will be used to assess the levels of each of these continuous parameters (IGA, PASI, BSA, DLQI, HADS, PEST, WPAI and PSD) over the five-year follow up period of the study.

For each visit, achievement of therapeutic endpoints, specifically IGA ≤ 1, IGA = 0, PASI 50, 75, 90 and 100 and DLQI ≤1 will be described with the proportion within each treatment cohort. Between-cohort differences will be assessed with generalized estimating equations. Time to achieving therapeutic endpoints, specifically IGA ≤ 1, IGA = 0, PASI 50, 75, 90 and 100 and DLQI ≤1, and the durability of response will be described with the Kaplan-Meier estimator. Between-cohort differences will be described with the log-rank test. Furthermore, Cox’s proportional hazards models that may adjust for potential confounders will be used to compare the two treatment cohorts.

### Informed consent

Novartis will provide to treating physicians or other involved medical professionals in a separate document a proposed informed consent form that complies with the Declaration of Helsinki principle and regulatory requirements and is considered appropriate for this study. The physician are instructed to keep the original informed consent form signed by the patient and a signed copy is also given to the patient.

Eligible patients may only be included in the study after providing written (witnessed, where required by law or regulation), IRB/EC-approved informed consent, or, if incapable of doing so, after such consent has been provided by a legally acceptable representative of the patient. In cases where the patient’s representative gives consent, the patient should be informed about the study to the extent possible given his/her understanding. If the patient is capable of doing so, he/she should assent by personally signing and dating the written informed consent document or a separate assent form. Informed consent must be obtained before any data are collected. The process of obtaining informed consent should be documented in the patient source documents.

## Discussion

Post-approval observational studies are the only source of information that allows ongoing rigorous prospective surveillance for safety signals under routine clinical care and the assessment of real-world effectiveness, which are necessary for establishing the true population-based benefit-risk ratio of approved treatments. Furthermore, when conducted properly, observational studies play an important role in understanding, and possibly driving, the physician decision-making process in real-world, including but not limited to, treatment selection, titration, and discontinuation, as well as rationale behind these decisions. Finally, the collection of real-world healthcare resource utilization data allows the health economic evaluation of available treatment options which is not possible in controlled clinical trial settings. The extensive clinical, health-economic, and patient-reported outcomes collected, the long-term patient follow-up, and the large sample size of PURE will allow the assessment of all aforementioned objectives. Furthermore, the limited inclusion/exclusion criteria and the observation of treatment cross-overs and treatment changes per the discretion of the treating physician, will provide a valid representation on the real-world management of patients with moderate to severe chronic plaque psoriasis. Finally, an important strength of the PURE study is the use of standardised validated questionnaires which increases the internal validity of the findings and allows the comparison with other similar studies.

An important limitation of the current study, inherent in observational studies, is a high likelihood of bias by indication due to the fact that patients will be treated per the discretion of the physician. However, itself, the potential description of such a bias, is of interest given that would represent the clinical reality. In order to minimize the effect of this bias on the study results, pertaining to the comparison of the two treatment cohorts, multivariate statistical methods will be used.

In summary, PURE is a large, prospective, disease registry that will provide an accurate and comprehensive overview of the clinical reality of treating moderate to severe chronic plaque psoriasis in Canada and Latin America which may have direct implications for patients, caregivers, and healthcare providers on the management of the illness, as well as for policy makers when assessing available interventions.

## Abbreviations

AE: Adverse Event
BSA: Body Surface Area
CI: Confidence Interval
DLQI: Dermatology Quality of Life Index
HADS: Hospital Anxiety and Depression Scale
HRU: Healthcare Resource Utilization
IGA: Investigator’s Global Assessment
IL: Interleukin
IRB: Institutional Review Board
MedDRA: Medical Dictionary for Regulatory Activities
PASI: Psoriasis Area and Severity Index (PASI)
PEST: Psoriasis Epidemiology Screening Tool
PSD: Psoriasis Symptom Diary
SAE: Serious Adverse Event
TNF: Tumor Necrosis Factor
UVA: Ultraviolet A
UVB: Ultraviolet B
WPAI: Work Productivity and Activity Impairment
